# Manufacture of Networks from Large Diameter Single-Walled Carbon Nanotubes of Particular Electrical Character

**DOI:** 10.3390/nano9040614

**Published:** 2019-04-14

**Authors:** Edyta Turek, Bogumila Kumanek, Slawomir Boncel, Dawid Janas

**Affiliations:** Department of Organic Chemistry, Bioorganic Chemistry and Biotechnology, Silesian University of Technology, B. Krzywoustego 4, 44-100 Gliwice, Poland; ea.turek@o2.pl (E.T.); Bogumila.Kumanek@polsl.pl (B.K.); Slawomir.Boncel@polsl.pl (S.B.)

**Keywords:** carbon nanotubes, electrical character, aqueous two-phase extraction

## Abstract

We have demonstrated that the aqueous two-phase extraction (ATPE) can differentiate between large diameter single-walled carbon nanotubes (CNTs) by electrical character. Introduction of “hydration modulators” to the ATPE machinery has enabled us to isolate metallic and semiconducting CNTs with ease. We have also shown that often there is a trade-off between the purity of the obtained fractions and the ability to separate both metallic and semiconducting CNTs at the same time. To isolate the separated CNTs from the matrices, we have proposed a method based on precipitation and hydrolysis, which can eliminate the need to use lengthy dialysis routines. In the final step, we prepared thin free-standing films from the sorted material and probed how electrical charge is transported through such macroscopic ensembles.

## 1. Introduction

The term carbon nanotubes (CNTs) may be somewhat misleading by implying that it is a plurality of exact copies of individual CNTs. However, the members of this population differ slightly from one another and these seemingly negligible discrepancies have a dramatic influence on the properties of the material. For instance, a diameter difference on the order of 0.1 Å is enough to make one CNT semiconducting and the other to have a metallic character [1,2]. The effect is valid not only for the electrical properties; their thermal [3,4], optical [5,6,7], and other attributes are also strongly affected. To tackle this problem, a spectrum of sorting methods has been established, which enable differentiation between CNTs of different types at the level of electrical character, chirality, or even handedness [8].

Large diameter single-walled carbon nanotubes (SWCNTs) are particularly attractive for various fields of science and technology, including photonics and microelectronics [9]. Their ability to emit light in the telecom range [10,11], as well as maintain high saturation current [12] at a reduced Schottky barrier [13], make them a very promising material. Unfortunately, with the increase in diameter, the differences between individual CNTs fade and sorting becomes challenging. Moreover, for some applications, macroscopic ensembles from defined CNTs such as films or fibers are necessary, but there are no straightforward techniques to reach this goal. The main obstacles are the lack of effective methods to obtain CNTs of selected electrical type at the large scale and, even if such sorted material is obtained, it can rarely be transformed into a CNT network free of contamination. A promising way to handle this problem was provided by He et al., who produced spontaneously aligned CNT films from a variety of parent materials [14].

In this contribution, we present our results of sorting large diameter SWCNTs by using a single-step adaptation [15] of the aqueous two-phase extraction (ATPE) method [16]. The key to reaching very high purity CNTs was to introduce a hydration modulator into the ATPE system, which enabled much more effective partitioning of semiconducting and metallic CNTs into top and bottom fractions, respectively. Large-diameter CNTs were conveniently divided into semiconducting and metallic rich fractions, and then they were transformed into thin free-standing films by using a method recently developed by us [17]. Characterization of the composition and electrical conductivity of such materials enabled us to validate whether the separation was successful. The developed methodology also demonstrated how to desorb the polymer species from the surface of the CNTs, which is a significant problem in the processing of sorted CNTs and which, to this day, is most commonly handled by lengthy dialysis.

## 2. Materials and Methods

Large-diameter single-walled carbon nanotubes (CNTs) of high purity with lengths exceeding 5 µm were purchased from OCSiAl (Tuball^TM^, Leudelange, Luxembourg) and purified by air treatment and reflux in HCl, according to a published method [18]. Purified CNTs (1 mg/mL) were dispersed in water using 20 g/L of sodium cholate (SC). The mixture was processed by a tip sonicator for 2 h (Hielscher UP200St, Teltow, Germany; 200 W, 50% amplitude, 50 mL, 2 h). Due to the evolution of heat over time, ice baths were used continuously to ensure proper homogenization of the material. Next, the dispersion was centrifuged at 11,000 rpm (Eppendorf 5804R centrifuge, Hamburg, Germany; 15,314 × g) for 2 h to remove the bundled CNTs from the dispersion (the top 80% of the supernatant volume was used for the study; the rest was discarded).

**Figure 1:**
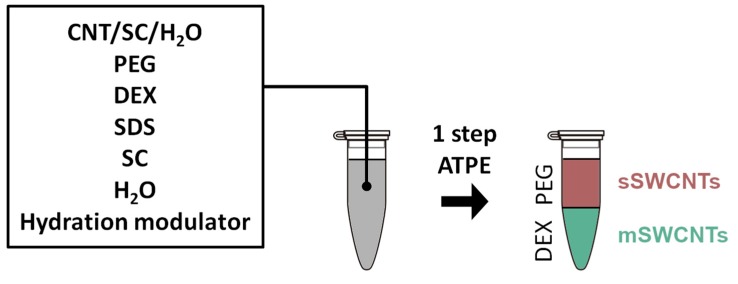
The method of separation used in the study—one-step aqueous two-phase extraction (ATPE), which, in this case, gives s-SWCNTs (semiconducting single-walled CNTs) and m-SWCNTs (metallic single-walled CNTs).

One-step separation by ATPE was executed by modifying an approach published by us [15] and that of Gui et al. [16] (by the introduction of a range of hydration modulators). CNT dispersion, Dextran (DEX, 20%, aq.), poly(ethylene glycol) (PEG, 50%, aq.), sodium cholate (SC, 10%, aq.), sodium dodecyl sulfate (SDS, 10%, aq.), hydration modulator (alanine, β-cyclodextrin, diethanolamine, hydrogen peroxide, ethylenediaminetetraacetic acid (EDTA), ethylene glycol, N,N-dimethylformamide, imidazole, poly(ethylene glycol) methyl ether (PEGme) Mw = 5,000 g/mol, polyvinylpyrrolidone (PVP), potassium persulfate, potassium phthalimide, sodium borohydride, sodium hypochlorite, thioacetamide, thiourea, or urea) and H_2_O were combined in an Eppendorf tube in the specified order (Figure 1) (exact experimental details are given in the Supplementary information file). The mixture was shaken vigorously until all the components were appropriately combined. Finally, to speed up the process of phase separation (which would otherwise take a few hours), the mixture was centrifuged for 3 min at 2,000 rpm (506 × g). After this time, the bottom DEX layer and the top PEG emerged spontaneously. The total volume was 1.53 mL each time.

Absorbance spectra were obtained from 400 to 1000 nm using Hitachi U-2910 spectrophotometer (Tokyo, Japan). Bottom and top phases were separated from each other and introduced to quartz cuvettes for measurements. Intensities were normalized to the global absorbance minimum which can be often found within or near the 700–800 nm range. Presented data were offset to improve the clarity of the presentation.

Scanning electron microscopy (SEM, FEI Nova NanoSEM, Hillsboro, OR, USA; 10 keV acquisition voltage) was employed to analyze the microstructure of the material.

To remove PEG and DEX, the following routines have been developed. Acetone was added to the PEG phase to precipitate the CNTs and the mixture was filtered under reduced pressure. CNTs were washed using warm distilled water (this step was repeated three times). The obtained solid was dried in a desiccator at room temperature to reach a constant weight. With regard to the DEX phase, 0.7 M HNO_3_ was used to precipitate the CNTs and the mixture was filtered under reduced pressure using warm distilled water, acetone, and methanol as the washing media. Then, Dextran was hydrolyzed by introducing the obtained solid into 1 M HCl solution, kept at 60 °C for 1 h under sonication. CNTs were separated by filtration under reduced pressure, washed with warm distilled water and subjected to another sonication step at 60 °C for 1 h, this time in distilled water. Finally, the obtained material was filtered under reduced pressure and washed with warm distilled water, acetone, and methanol. After the process, the product was dried in a desiccator at room temperature to reach a constant weight.

Thermograms (Linseis TA system, Selb, Germany) were acquired in the flow of air (50 mL/min) at a 10 °C/min heating rate. A total of 1.5 mg of material was used for each measurement.

To make the films, a previously reported routine was employed [17]. In brief, one equivalent of CNTs was combined with one equivalent of ethyl cellulose (both 1 wt% with respect to the solvent) in acetone/toluene mixture (1:1, V/V). Then, the mixture kept in an ice bath was sonicated to obtain a uniform dispersion. Subsequently, the CNT paint was deposited onto a Kapton^®^ film (RS Components, Corby, UK), detached from its surface and annealed in air to remove the ethyl cellulose. CNT films produced this way (using unsorted CNTs or predominantly metallic/semiconducting) were further characterized.

Raman spectroscopy (inVia Renishaw Raman microscope, Wotton-under-Edge, UK; λ = 514, 633, or 780 nm, where indicated) was used to record inelastic scattering from the samples within a 100 to 3200 cm^−1^ range. To lower the effect of background, 50 accumulations were acquired for each sample.

Electrical resistivity was measured by using a four-probe method (Keithley 2450, Cleveland, OH, USA). The values were recorded at room temperature for neat and p-doped films (BF_3_ solution in methanol was dripped onto the film and the solvent was allowed to evaporate).

## 3. Results and Discussion

### 3.1. Characterization of the Material

**Figure 2:**
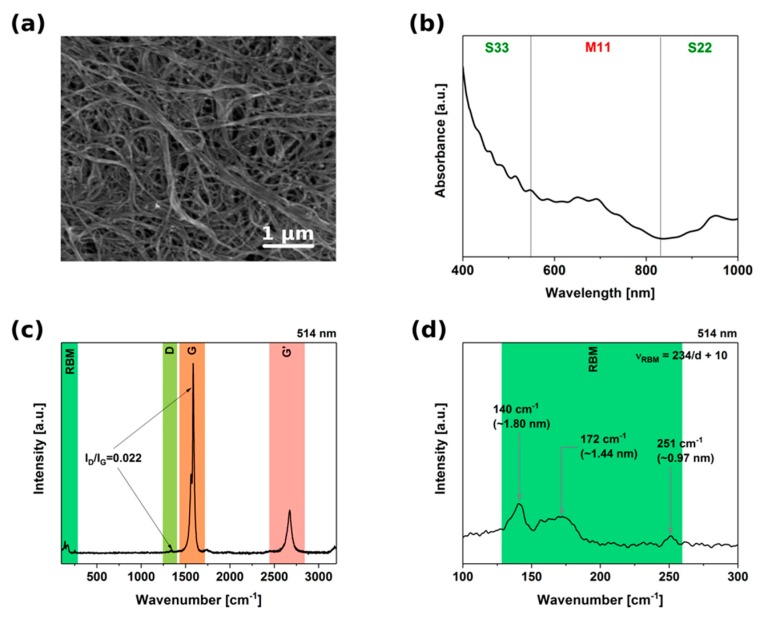
Characterization of the starting material. (**a**) Scanning Electron Microscopy (SEM) micrograph, (**b**) absorbance spectrum, (**c**) Raman spectrum, (**d**) close-up plot of Radial Breathing Mode (RBM) area from the Raman spectrum (514 nm excitation).

Sorting of CNTs is based on the principle that various CNT structures have slightly dissimilar interactions with the environment based on the minute differences in their structure. As a consequence, any type of unwanted functionalization can not only influence the course of separation, but after reaching a certain threshold, it makes the differentiation impossible. First, we confirmed that the selected material was suitable for the study by characterization of its microstructure and composition (Figure 2). The presence of non-CNT macroscopic adulterants was minimal, as observed under SEM. What is more, the CNTs were very much bundled-up as expected for single-walled CNTs.

The absorbance spectra showed signals both in the M_11_ and S_22_/S_33_ areas, indicating that the unsorted material was indeed composed of a mixture of CNTs of different electrical character. Furthermore, characterization of the surface by Raman spectroscopy confirmed high purity of the material. I_D_/I_G_, indicative of the level of crystal imperfection (D band—sp^3^ impurities, G band—sp^2^ graphitic lattice), was as low as 0.022. Additionally, splitting of the G band into G+ and G− components could be detected. It is important to note that no defects were introduced even during prolonged sonication of the material to make a CNT dispersion (Figure S1). Analysis of the Radial Breathing Mode (RBM) area confirmed the large-diameter character of the material. According to the manufacturer, the diameter distribution of this material is within the 1.8 ± 0.4 nm range. We detected CNTs with diameters from about 1 nm up to 1.87 nm by recalculating the wavenumbers to diameter [19,20,21] (please refer to Figure S2 to see Raman spectra acquired at 633 and 780 nm). Raman spectroscopy is a resonant method of characterization, so only those CNTs which are in tune with the wavelength of the lasers are detected (the rest remains invisible). Secondly, the peaks are Lorentzian in nature, so wavenumber of their maxima should be taken into consideration.

Therefore, in light of these results we can claim that the diameter of the material used for the study was within the 1–2 nm range.

### 3.2. Regular ATPE Separation

**Figure 3:**
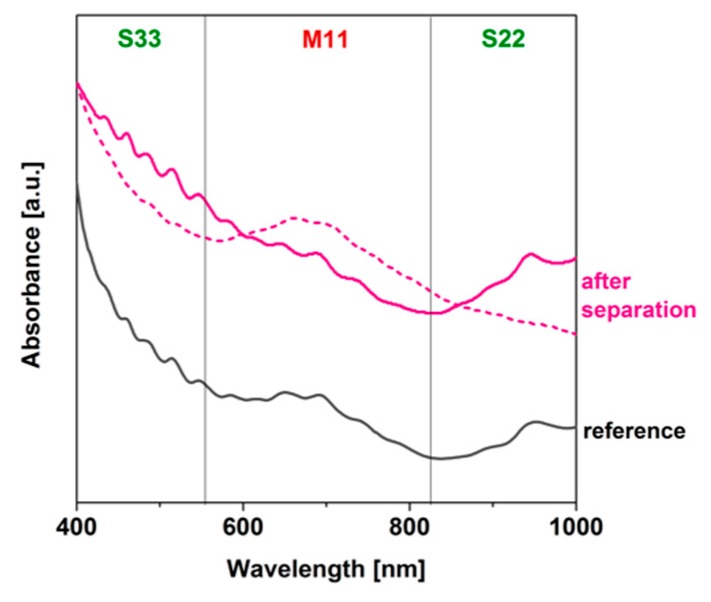
ATPE of large-diameter single-walled carbon nanotubes (CNTs) without addition of hydration modulators. Dextran-rich bottom phase (dashed line), PEG-rich top phase (solid line). Reference dispersion shown in black.

First, we wanted to find out how the ATPE works on large-diameter single-walled CNTs without introducing any hydration modulator (Figure 3).

Preliminary results were encouraging, as we managed to separate a fraction significantly enriched with the metallic type of CNTs in the PEG phase. The peak of absorbance emerged in the M_11_ range and the intensity of the signal coming from the neighboring S_33_ and S_22_ areas [19] was significantly reduced as compared with the parent material. On the other hand, the bottom phase did not show an appropriate degree of isolation of the corresponding semiconducting fraction, as it resembled the reference. It was clear that the system had to be tuned to reach simultaneous preference towards these types of CNTs by the two phases and hence isolate them from each other. We decided to introduce another component into the ATPE system, which could influence how the surfactants encapsulating the CNTs arrange themselves on their surface.

### 3.3. Effect of H_2_O_2_ Addition

**Figure 4:**
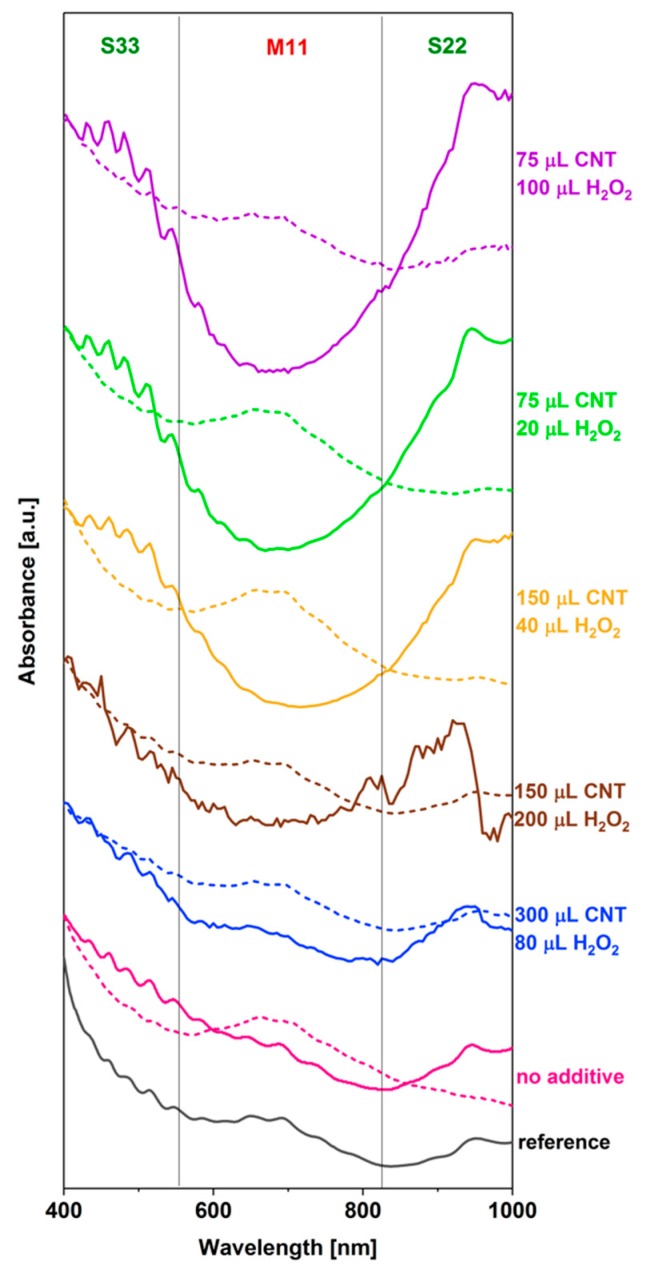
The influence of the volume of the CNT dispersion and hydration modulating agent (H_2_O_2_) in the ATPE system on the course of the separation. Dextran-rich bottom phase (dashed line), PEG-rich top phase (solid line).

The first compound that we decided to exploit for this purpose was hydrogen peroxide because of its high compatibility with the ATPE components and well-documented electronic interactions with carbon nanostructures [22,23,24] (Figure 4). At a high CNT loading of 300 μL with added 80 μL of 30 wt% H_2_O_2_, the separation attempt was not successful. The excess of metallic CNTs was lower than in the reference sample and again we did not manage to separate the semiconducting ones from the mixture. We decided to lower the content of CNTs to 150 μL and tried introducing high (200 μL) or low (40 μL) content of H_2_O_2_.

**Figure 5:**
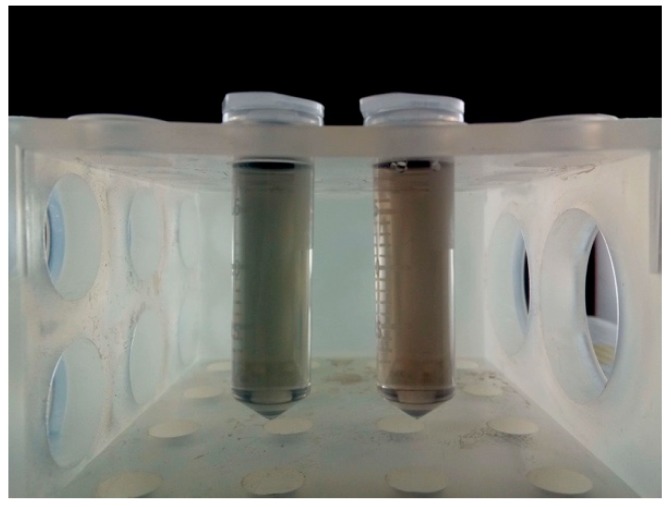
Photograph of the two phases after separation into CNT dispersions, composed predominantly of metallic (left) and semiconducting (right) species.

To our delight, the differentiation of these two CNT types was achieved in the latter case. Not only was the peak of metallic CNTs much more defined than when no additive was employed, but also the semiconducting CNTs were obtained. Because of our previous experience, which suggests that, often, lowering the relative content of CNTs in the ATPE mixture improves the selectivity, we decided to lower the starting CNT amount by half, down to 75 μL, and also added 20 μL of H_2_O_2_. Unfortunately, as can be seen in the spectrum of the semiconducting-rich fraction, the intensity of interband metallic transitions increased slightly between 550 and 650 nm. Simultaneously, the peak of the corresponding metallic-rich fraction became less pronounced, which indicated that some of the metallic CNTs migrated to the top phase. Finally, and interestingly, by increasing the amount of added H_2_O_2_ to 100 μL, one could obtain the highly semiconducting fraction in the top phase, but a significant part of metallic CNTs would be lost from the bottom phase and shifted to the interface. This is completely opposite of the results from the neat ATPE, wherein only the metallic fraction was obtained. Closer investigation of the spectra clearly showed a trade-off between the resolution of the system toward a particular CNT type and a high yield of separation. When both metallic and semiconducting fractions were isolated to the respective phases, the purity could be high, but not necessarily the highest possible. Further enrichment in the metallic or semiconducting content was possible at the expense of purity of the complementary phase. We can conclude that the optimum parameters to obtain the highest amount of metallic and semiconducting species in one experiment are 150 μL CNT and 40 μL H_2_O_2_. Figure 5 depicts what these two phases look like after separation.

### 3.4. Effect of Poly(Ethylene Glycol) Methyl Ether Addition

**Figure 6:**
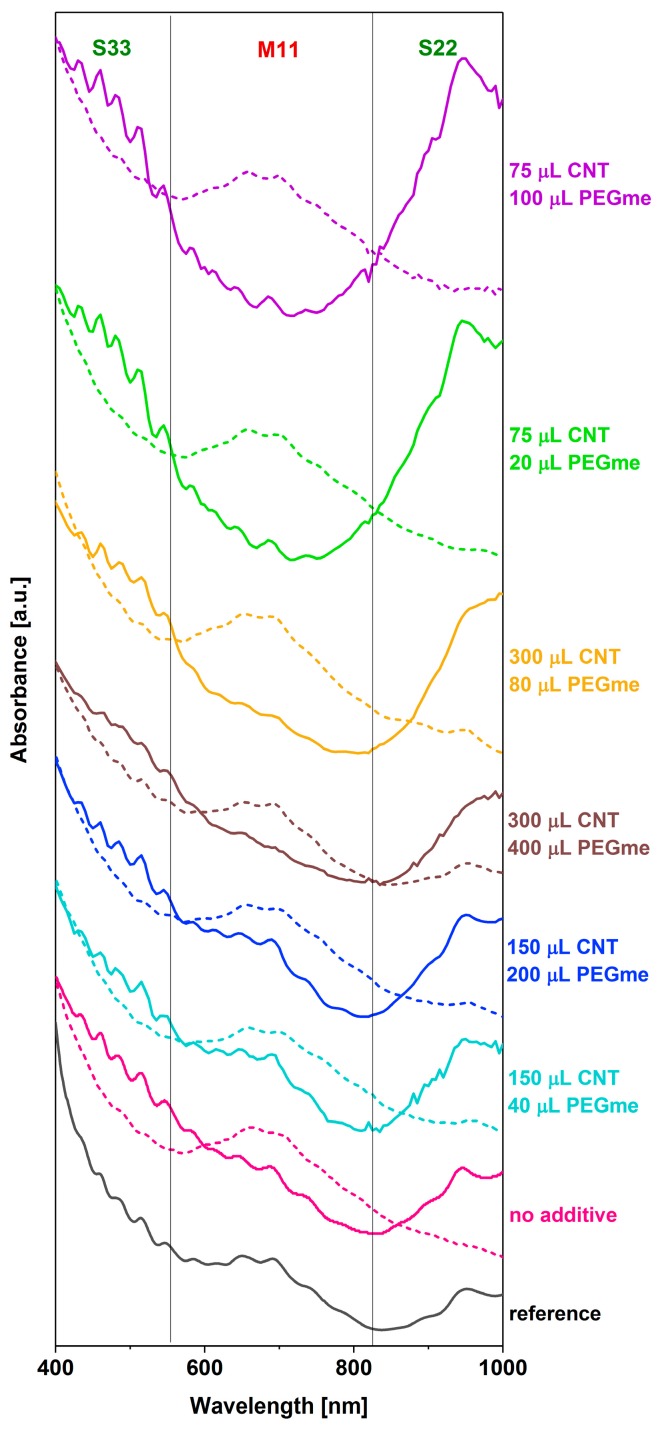
The influence of the volume of the CNT dispersion and hydration modulating agent (PEGme) in the ATPE system on the course of the separation. Dextran-rich bottom phase (dashed line), PEG-rich top phase (solid line).

More complicated structures than H_2_O_2_ can also fine tune the metallic–semiconducting separation ability of the ATPE. Poly(ethylene glycol) methyl ether (PEGme) also enabled us to significantly enrich the purity of the metallic and semiconducting CNT fractions (Figure 6).

Again, the best results were obtained when a relatively small amount of CNTs was introduced into the ATPE system (ca. 75 μL). In this case, however, the contamination of semiconducting fractions with the metallic CNTs was more obvious at this level. Only when the volume of the CNT dispersion and PEGme was increased four-fold to 300 and 80 μL, respectively, the purity was improved, but again at the expense of the yield of separation (the desired decrease in the M_11_ range of the semiconducting fraction was accompanied by a loss of intensity in the S_22_ zone). Other combinations of parameters involving high PEGme content were much less successful than the aforementioned conditions. It appears that PEGme, because of its hydrophobic methyl head, can somehow change the interaction of the CNTs with the ATPE matrix. Simple modification of the PEG content did not lead to the same results.

### 3.5. Effect of Addition of Other Hydration Modulators

We tried a wide range of compounds as hydration modulators (please refer to the Supplementary information file for all the conditions employed). Firstly, introducing redox active inorganic compounds, such as NaBH_4_ (Figure S3), K_2_S_2_O_8_ (Figure S4), or NaClO (Figure S5), did not lead to any separation at all, as most of the CNTs aggregated at the interface. Secondly, we explored organic compounds with and without heteroatoms in their structure (Figures S6–S17, ethylene glycol, alanine, diethanolamine, ethylenediaminetetraacetic acid (EDTA), urea, dimethylformatide (DMF), phthalimide, imidazole, thiourea, thioacetamide, β-cyclodextrin, and polyvinylpyrrolidone (PVP)). Either no improvement was observed, or the shape of the spectra did not resemble CNTs enriched with particular electrical character. In fact, addition of certain chemical species resulted in the collapse of the ATPE system or lack of clarity of the constituting phases. From our experience so far, the introduction of only the two aforementioned species (H_2_O_2_ and PEGme) had a positive influence on the course of separation.

### 3.6. Removal of ATPE Remnants

**Figure 7:**
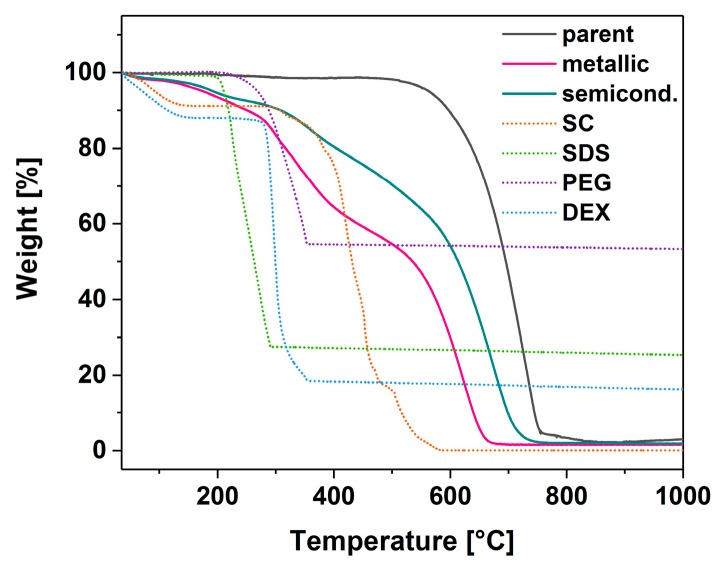
Thermograms of parent and sorted material as well as those of ATPE components.

We carried out a large scale separation (100 fold increase—total volume of 153 mL) of CNTs to isolate both semiconducting and metallic fractions at the highest possible purity in one go (relative parameters: 150 μL CNT and 40 μL H_2_O_2_) for further examination. In the first step, we developed a process of isolation of these species from PEG and DEX matrices, respectively. Usually, this is accomplished by a lengthy dialysis, which requires high pressure and expensive membranes. Our chemical approach was designed to precipitate out the selected material and hydrolyze certain chemical compounds to enable a simple and convenient separation from the CNTs by filtration under reduced pressure. As shown in the thermograms, both metallic- and semiconducting-rich fractions were essentially purified from PEG and DEX (Figure 7). It should be noted, however, that the final samples contained a certain amount of SC, which was used for dispersion at the very beginning. Oxidation of the material took place at relatively high temperatures for single-walled CNTs (ca. 600 °C), but this can be justified by taking into the consideration their large diameter.

### 3.7. Sorting Outcome Characterization by Raman Spectroscopy

**Figure 8:**
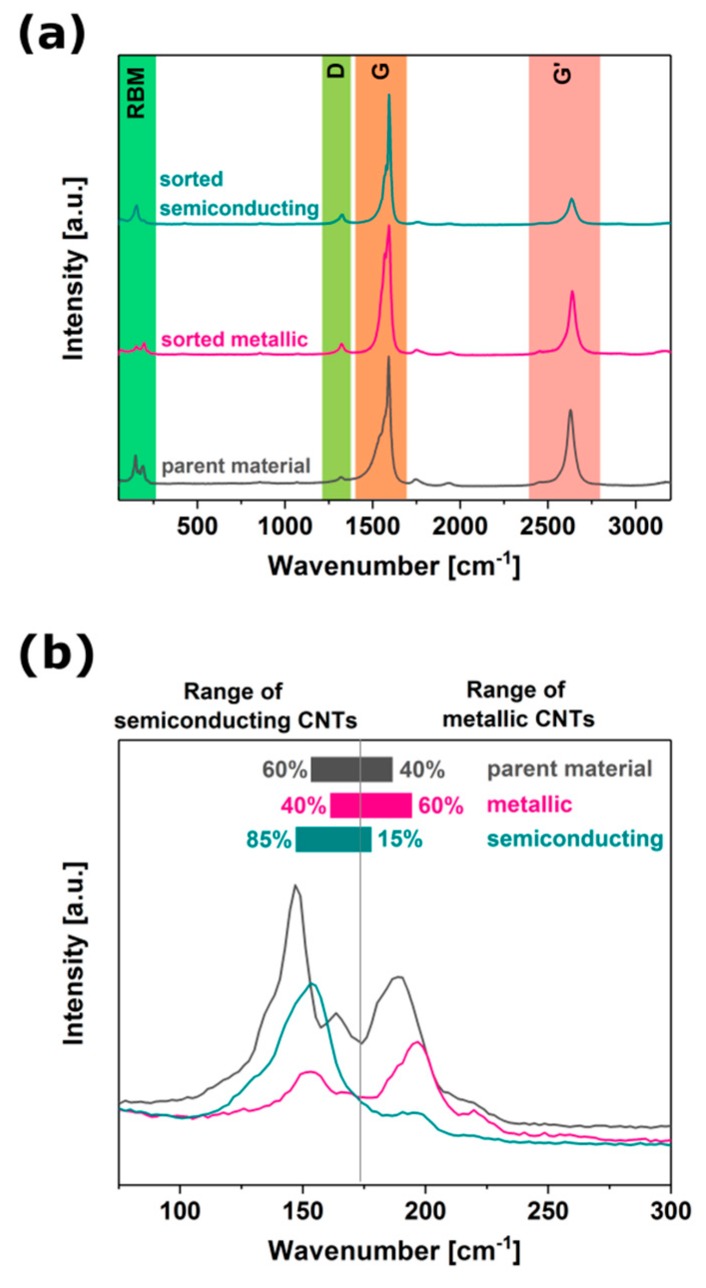
Raman spectra: (**a**) Overview of all the peaks, (**b**) magnification of RBM area.

To get a better insight into the separation, we studied the obtained samples by Raman spectroscopy (Figure 8). It can be seen that enrichment of the samples with particular electrical character was successful, although it led to complete differentiation between these two CNT families. The parent material was 60% semiconducting and 40% metallic. Separation by modified ATPE resulted in manufacture of the material having 60% metallic character or 85% semiconducting character of the bottom and top phases, respectively. It should be noted that the Raman technique is a resonant process, therefore the conclusions are valid only for the specified wavelength (633 nm). As a consequence, the observed change in RBM intensities should be considered as a qualitative measure of the observed phenomena, since there are more precise methods for appropriate quantification. According to the absorbance spectra presented earlier, the extent of the separation may be much larger because, under certain conditions, the metallic fraction does not seem to contain semiconducting CNTs and vice versa.

### 3.8. Electrical Conductivity of Sorted CNT Films

**Figure 9:**
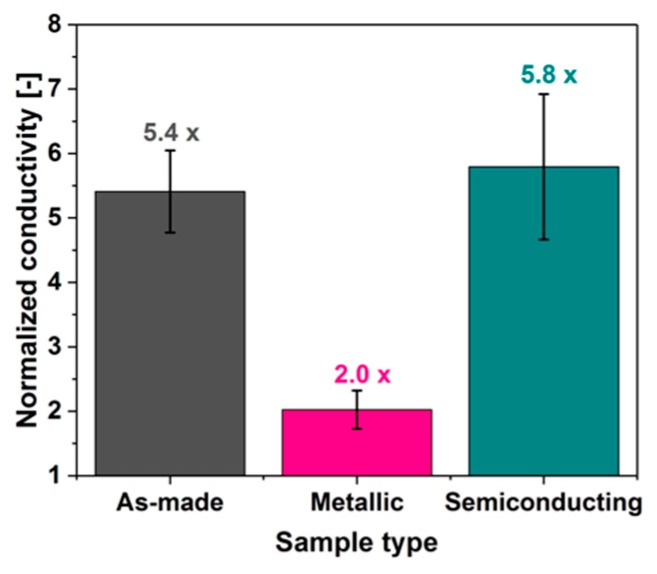
The influence of BF_3_ on the electrical conductivity of unsorted and sorted CNT films.

Finally, we wanted to gauge the electrical performance of these materials (Figure 9). To accomplish this goal, we created thin free-standing films from them and characterized how their electrical conductivity (normalized to unity to eliminate uncertainties in the determination of samples dimensions) responds to doping. BF_3_ was selected as a doping agent because of its very strong influence on the electrical conductivity of CNTs [25]. Addition of BF_3_ in methanol to the film from as-made unsorted SWCNTs caused almost a five-fold increase in electrical conductivity. Very similar results were obtained in the case of the semiconducting-rich film, whereas the film composed predominantly of metallic CNTs experienced only a two-fold reduction in electrical resistance.

This proves our earlier suspicion that the large-diameter SWCNT material Tuball^TM^ is of predominantly semiconducting character [25]. BF_3_ improves electrical conductivity most probably by introducing an impurity band to the density of states (DOS) similarly to the action of interhalogen compounds [1], which leads to lowering the bandgap of semiconducting CNTs. The reason why semiconducting-rich CNT films did not show an even larger increase in conductivity (as compared with unsorted samples) may be explained by the fact that the electronic transport in these materials is often limited by the contact resistance between individual CNTs, or caused by other extrinsic factors such as CNT misalignment or presence of impurities, as recently reported by Bulmer et al. [26].

On the other hand, electrical conductivity of metallic-rich CNT films doubled. As indicated by the Raman spectra, these films are adulterated with a minor amount of semiconducting CNTs, which can respond to BF_3_ doping. This suggests that the percolation pathway in this case may not be constructed exclusively from metallic CNTs, but “bridges” exist, which are made of semiconducting CNTs. An increase in electrical conductivity of these semiconducting CNTs could then be responsible for the increase in the overall electrical conductivity of the network. One could also consider that the newly introduced impurity band increases the density of states near the Fermi energy, which is otherwise low for intrinsic metallic CNTs. Doping could then increase the Fermi energy beyond M_11_ and improve the electrical conductivity of the network composed predominantly of metallic CNTs.

It should also be considered that, when the doping agent is introduced to a CNT by using a volatile solvent as a vector, it often causes some densification of the material [1]. As a consequence, contact between the constituting CNTs is improved and the resistance is reduced. This could explain why the conductivity of metallic-rich CNT films improved upon exposure of the material to BF_3_. In such a scenario, improvement to the electrical conductivity is caused by two factors: Reduction of intrinsic electrical resistance of semiconducting CNTs and optimization of geometrical factors, which alleviate the problem of contact resistance (the extrinsic contribution to the electrical transport).

## 4. Conclusions

We presented how large-diameter SWCNTs can be sorted according to the electrical character by using the ATPE method. Introduction of hydration modulators (H_2_O_2_ and PEGme) very much improved the resolution of the one-step system, which we developed. The obtained fractions were predominantly metallic or semiconducting, as observed by both absorbance and Raman spectroscopy. After isolation of these species from the corresponding matrices using our approach based on precipitation and hydrolysis, we manufactured thin free-standing films from them. Although surfactant was still detected on the surface of CNTs, this technique was more rapid for removal of Dextran or PEG than common hydrolysis. The doping tests indicated the way the electrical charge is transported through such (un)sorted CNT ensembles. In parallel, the electrical experiments manifested once again the problem of contact resistance—one of the most serious issues for CNT fibers and films, which must be taken care of to implement them in real life.

## Supplementary Materials

The following are available online at https://www.mdpi.com/2079-4991/9/4/614/s1, Detailed description of parameters used for the preparation of the material (CNT dispersion and hydration modulator solutions), Figure S1: Raman spectra of the parent CNT material before and after sonication, Figure S2: Raman spectra of CNT dispersions acquired at 633 nm and 780 nm laser excitation wavelength, Table S1: ATPE parameters for the experiments with H_2_O_2_ as hydration modulator, Table S2: ATPE parameters for the experiments with PEG as hydration modulator, Figure S3: Absorbance spectra for the experiments with NaBH_4_ as hydration modulator, Table S3: ATPE parameters for the experiments with NaBH_4_ as hydration modulator, Figure S4: Absorbance spectra for the experiments with K_2_S_2_O_8_ as hydration modulator, Table S4: ATPE parameters for the experiments with K_2_S_2_O_8_ as hydration modulator, Figure S5: Absorbance spectra for the experiments with NaClO as hydration modulator, Table S5: ATPE parameters for the experiments with NaClO as hydration modulator, Figure S6: Absorbance spectra for the experiments with ethylene glycol as hydration modulator, Table S6: ATPE parameters for the experiments with ethylene glycol as hydration modulator, Figure S7: Absorbance spectra for the experiments with alanine as hydration modulator, Table S7: ATPE parameters for the experiments with alanine as hydration modulator, Figure S8: Absorbance spectra for the experiments with diethanolamine as hydration modulator, Table S8: ATPE parameters for the experiments with diethanolamine as hydration modulator, Figure S9: Absorbance spectra for the experiments with ethylenediaminetetraacetic acid as hydration modulator, Table S9: ATPE parameters for the experiments with ethylenediaminetetraacetic acid as hydration modulator, Figure S10: Absorbance spectra for the experiments with urea as hydration modulator, Table S10: ATPE parameters for the experiments with urea as hydration modulator, Figure S11: Absorbance spectra for the experiments with *N,N*-dimethylformamide as hydration modulator, Table S11: ATPE parameters for the experiments with *N,N*-dimethylformamide as hydration modulator, Figure S12: Absorbance spectra for the experiments with phthalimide as hydration modulator, Table S12: ATPE parameters for the experiments with phthalimide as hydration modulator, Figure S13: Absorbance spectra for the experiments with imidazole as hydration modulator, Table S13: ATPE parameters for the experiments with imidazole as hydration modulator, Figure S14: Absorbance spectra for the experiments with thiourea as hydration modulator, Table S14: ATPE parameters for the experiments with thiourea as hydration modulator, Figure S15: Absorbance spectra for the experiments with thioacetamide as hydration modulator, Table S15: ATPE parameters for the experiments with thioacetamide as hydration modulator, Figure S16: Absorbance spectra for the experiments with *β*-cyclodextrin as hydration modulator, Table S16: ATPE parameters for the experiments with *β*-cyclodextrin as hydration modulator, Figure S17: Absorbance spectra for the experiments with polyvinylpyrrolidone as hydration modulator, Table S17: ATPE parameters for the experiments with polyvinylpyrrolidone as hydration modulator.

## References

[1] Janas D., Milowska K.Z., Bristowe P.D., Koziol K.K. (2017). Improving the electrical properties of carbon nanotubes with interhalogen compounds. Nanoscale.

[2] Zhang M., Li J. (2009). Carbon nanotube in different shapes. Mater. Today.

[3] Kumanek B., Janas D. (2019). Thermal conductivity of carbon nanotube networks—Review. J. Mater. Sci..

[4] Mir M., Ebrahimnia-Bajestan E., Niazmand H., Mir M. (2012). A novel approach for determining thermal properties of single-walled carbon nanotubes. Comput. Mater. Sci..

[5] Kataura H., Kumazawa Y., Maniwa Y., Umezu I., Suzuki S., Ohtsuka Y., Achiba Y. (1999). Optical properties of single-wall carbon nanotubes. Synth. Met..

[6] Sfeir M.Y., Beetz T., Wang F., Huang L.M., Huang X.M.H., Huang M.Y., Hone J., O'Brien S., Misewich J.A., Heinz T.F. (2006). Optical spectroscopy of individual single-walled carbon nanotubes of defined chiral structure. Science.

[7] Janas D., Czechowski N., Krajnik B., Mackowski S., Koziol K.K. (2013). Electroluminescence from carbon nanotube films resistively heated in air. Appl. Phys. Lett..

[8] Janas D. (2018). Towards monochiral carbon nanotubes: A review of progress in the sorting of single-walled carbon nanotubes. Mater. Chem. Front..

[9] Yang D.H., Hu J.W., Liu H.P., Li S.L., Su W., Li Q., Zhou N.G., Wang Y.C., Zhou W.Y., Xie S.S. (2017). Structure Sorting of Large-Diameter Carbon Nanotubes by NaOH Tuning the Interactions between Nanotubes and Gel. Adv. Funct. Mater..

[10] He X.W., Htoon H., Doorn S.K. (2017). Tunable Room-Temperature Single-Photon Emission at Telecom Wavelengths from sp3 Defects in Carbon Nanotubes. Nat. Photonics.

[11] Graf A., Zakharko Y., Schiessl S.P., Backes C., Pfohl M., Flavel B.S., Zaumseil J. (2016). Large scale, selective dispersion of long single-walled carbon nanotubes with high photoluminescence quantum yield by shear force mixing. Carbon.

[12] Tulevski G.S., Franklin A.D., Frank D., Lobez J.M., Cao Q., Park H., Afzali A., Han S.J., Hannon J.B., Haensch W. (2014). Toward High-Performance Digital Logic Technology with Carbon Nanotubes. ACS Nano.

[13] Chen Z.H., Appenzeller J., Knoch J., Lin Y.M., Avouris P. (2005). The role of metal-nanotube contact in the performance of carbon nanotube field-effect transistors. Nano Lett..

[14] He X., Gao W., Xie L., Li B., Zhang Q., Lei S., Robinson J.M., Haroz E.H., Doorn S.K., Wang W. (2016). Wafer-scale monodomain films of spontaneously aligned single-walled carbon nanotubes. Nat. Nanotechnol..

[15] Turek E., Shiraki T., Shiraishi T., Shiga T., Fujigaya T., Janas D. (2019). Single-step isolation of carbon nanotubes with narrow-band light emission characteristics. Sci. Rep..

[16] Gui H., Streit J.K., Fagan J.A., Walker A.R.H., Zhou C.W., Zheng M. (2015). Redox Sorting of Carbon Nanotubes. Nano Lett..

[17] Janas D., Rdest M., Koziol K.K.K. (2017). Free-standing films from chirality-controlled carbon nanotubes. Mater. Des..

[18] Clancy A.J., White E.R., Tay H.H., Yau H.C., Shaffer M.S.P. (2016). Systematic comparison of conventional and reductive single-walled carbon nanotube purifications. Carbon.

[19] Fagan J.A., Haroz E.H., Ihly R., Gui H., Blackburn J.L., Simpson J.R., Lam S., Walker A.R.H., Doorn S.K., Zheng M. (2015). Isolation of > 1 nm Diameter Single-Wall Carbon Nanotube Species Using Aqueous Two-Phase Extraction. ACS Nano.

[20] Souza A.G., Chou S.G., Samsonidze G.G., Dresselhaus G., Dresselhaus M.S., An L., Liu J., Swan A.K., Unlu M.S., Goldberg B.B. (2004). Stokes and anti-Stokes Raman spectra of small-diameter isolated carbon nanotubes. Phys. Rev. B.

[21] Fantini C., Jorio A., Souza M., Strano M.S., Dresselhaus M.S., Pimenta M.A. (2004). Optical transition energies for carbon nanotubes from resonant Raman spectroscopy: Environment and temperature effects. Phys. Rev. Lett..

[22] Goran J.M., Phan E.N.H., Favela C.A., Stevenson K.J. (2015). H_2_O_2_ Detection at Carbon Nanotubes and Nitrogen-Doped Carbon Nanotubes: Oxidation, Reduction, or Disproportionation?. Anal. Chem..

[23] Miao Z.Y., Zhang D., Chen Q. (2014). Non-enzymatic Hydrogen Peroxide Sensors Based on Multi-wall Carbon Nanotube/Pt Nanoparticle Nanohybrids. Materials.

[24] Xie A.J., Liu Q.X., Ge H.L., Kong Y. (2015). Novel H_2_O_2_ electrochemical sensor based on graphene-polyacrylamide composites. Mater. Technol..

[25] Janas D. (2018). Powerful doping of chirality-sorted carbon nanotube films. Vacuum.

[26] Bulmer J.S., Gspann T.S., Orozco F., Sparkes M., Koerner H., Di Bernardo A., Niemiec A., Robinson J.W.A., Koziol K.K., Elliott J.A. (2017). Photonic Sorting of Aligned, Crystalline Carbon Nanotube Textiles. Sci. Rep..

