# Supplementary material for: Manufacture of Networks from Large Diameter Single-Walled Carbon Nanotubes of Particular Electrical Character
Source: Nanomaterials (Basel). 2019 Apr 14;9(4):614. doi: 10.3390/nano9040614 (PMC6523666; doi:10.3390/nano9040614)
Supplement: Supplementary file 1 [file nanomaterials-09-00614-s001.pdf]

Electronic supplementary information

Manufacture of networks from large diameter single-walled carbon nanotubes of particular electrical character

Edyta Turek[a], Bogumiła Kumanek[a], Sławomir Boncel[a], Dawid Janas[a,*],

1. Experimental

Single-walled carbon nanotubes (SWCNTs, Tuball™) were obtained from OCSiAl (1.8±0.4 nm) and purified according to a published methodology (air treatment with subsequent reflux in HCl)[1]. The CNTs were dispersed in H_2O (1 mg/mL concentration) by sonication (Hielscher UP50H) in the presence of SC (2%) for 2 hours. During the treatment, the material was kept at low temperature (ice-bath) to improve the quality of the dispersion. Next, the SWCNT dispersion was centrifuged at 11,000 rpm (Eppendorf 5804R centrifuge) for 2h to sediment the non-individualized CNTs. Upper 80% of the supernatant was separated and used for the study.

Dextran (DEX) Mw=70,000 g/mol, poly(ethylene glycol) (PEG) Mw=6,000 g/mol, sodium dodecyl sulphate (SDS), sodium cholate (SC) and hydration modulators: Alanine, β-cyclodextrin, diethanolamine, hydrogen peroxide, ethylenediaminetetraacetic acid (EDTA), ethylene glycol, N,N-dimethylformamide, imidazole, poly(ethylene glycol) methyl ether (PEGme) Mw=5,000 g/mol, polyvinylpyrrolidone (PVP), potassium persulfate, potassium phthalimide, sodium borohydride, sodium hypochlorite, thioacetamide, thiourea and urea were of analytical quality. When the substance was in a solid form, a 10%wt solution in distilled water was prepared.

2. Additional Raman spectra

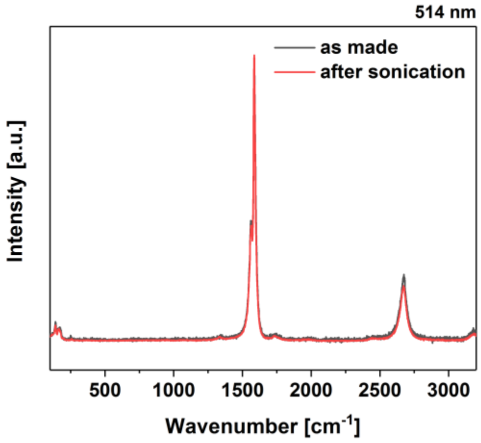

Figure S1. Raman spectra of the parent material before and after sonication.

[1] A.J. Clancy, E.R. White, H.H. Tay, H.C. Yau, M.S.P. Shaffer, Systematic comparison of conventional and reductive single-walled carbon nanotube purifications, Carbon 108 (2016) 423-432.

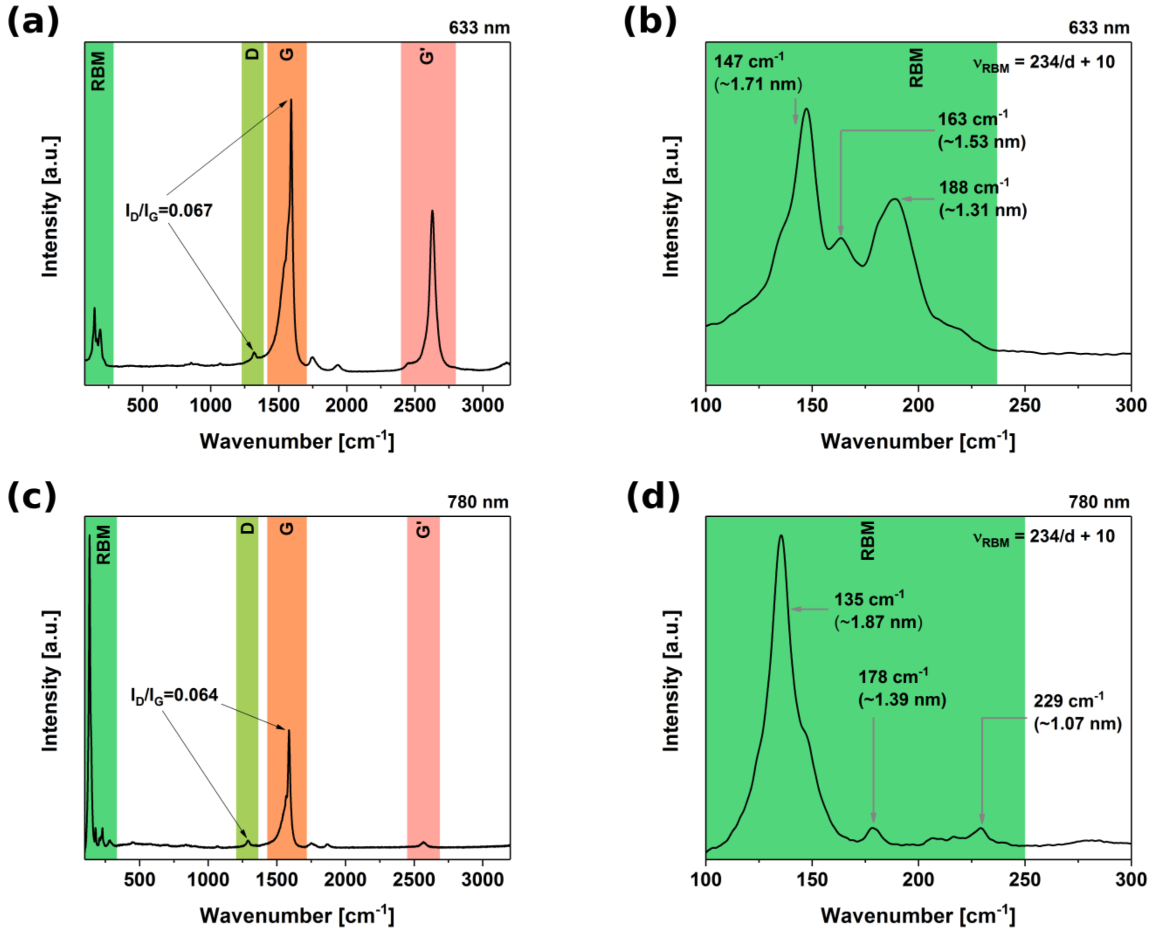

Figure S2. Raman spectra with magnification of the corresponding RBM areas for (a,b) 633 nm and (c,d) 780 nm laser excitation wavelength.

3. Separation parameters and absorbance data

3.1. H_2O_2 as hydration modulator

Table S1. ATPE parameters (H_2O_2 as hydration modulator)

	Volume [µL]						
	**CNT 1mg/mL**	**DEX 20%wt**	**PEG 50%wt**	**SC 10%wt**	**SDS 10%wt**	**H_2O_2 30%wt**	**H_2O**
No additive	75	450	180	120	60	–	645
1.	300	450	180	120	60	80	340
2.	150	450	180	120	60	200	370
3.	150	450	180	120	60	40	530
4.	75	450	180	120	60	20	625
5.	75	450	180	120	60	100	545

3.2. PEG as hydration modulator

Table S2. ATPE parameters (PEG as hydration modulator)

	Volume [µL]						
	**CNT 1mg/mL**	**DEX 20%wt**	**PEG 50%wt**	**SC 10%wt**	**SDS 10%wt**	**PEG 10%wt**	**H_2O**
No additive	75	450	180	120	60	–	645
1.	150	450	180	120	60	40	530
2.	150	450	180	120	60	200	370
3.	300	450	180	120	60	400	20
4.	300	450	180	120	60	80	340
5.	75	450	180	120	60	20	625
6.	75	450	180	120	60	100	545

3.3. Inorganic compounds as hydration modulators

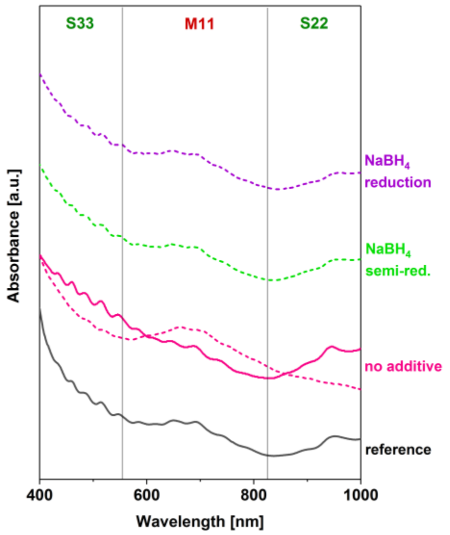

Figure S3 Absorbance spectra of bottom (dashed line) and top (solid line) phases after ATPE carried out in the indicated conditions (with and without $NaBH_4$ addition). Missing spectra did not reveal the presence of CNTs.

Table S3. ATPE parameters ($NaBH_4$ as hydration modulator)

	Volume [µL]						
	**CNT 1mg/mL**	**DEX 20%wt**	**PEG 50%wt**	**SC 10%wt**	**SDS 10%wt**	**$NaBH_4$ 10%wt**	**H_2O**
No additive	75	450	180	120	60	–	645
Semi-reduced	75	450	180	120	60	105	540
Fully-reduced	75	450	180	120	60	300	345

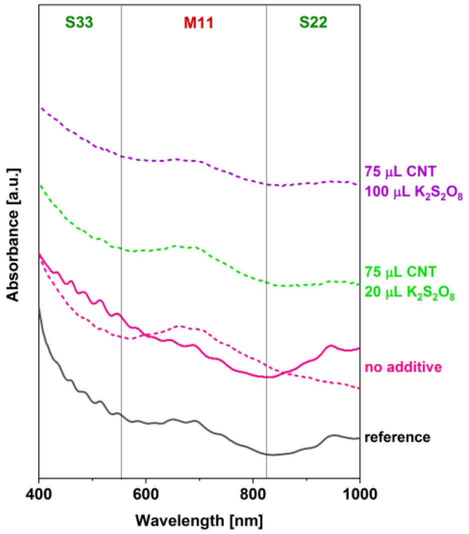

Figure S4 Absorbance spectra of bottom (dashed line) and top (solid line) phases after ATPE carried out in the indicated conditions (with and without $K_2S_2O_8$ addition). Missing spectra did not reveal the presence of CNTs.

Table S4. ATPE parameters ($K_2S_2O_8$ as hydration modulator)

	**Volume [µL]**						
	**CNT 1mg/mL**	**DEX 20%wt**	**PEG 50%wt**	**SC 10%wt**	**SDS 10%wt**	**$K_2S_2O_8$ 10%wt**	**H_2O**
No additive	75	450	180	120	60	–	645
Low-content	75	450	180	120	60	20	625
High-content	75	450	180	120	60	100	545

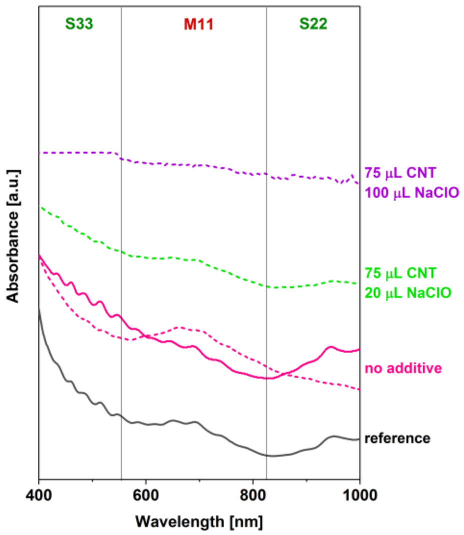

Figure S5 Absorbance spectra of bottom (dashed line) and top (solid line) phases after ATPE carried out in the indicated conditions (with and without NaClO addition). Missing spectra did not reveal the presence of CNTs.

Table S5. ATPE parameters (NaClO as hydration modulator)

	**Volume [µL]**						
	**CNT 1mg/mL**	**DEX 20%wt**	**PEG 50%wt**	**SC 10%wt**	**SDS 10%wt**	**NaClO 10%wt**	**H_2O**
No additive	75	450	180	120	60	–	645
Low-content	75	450	180	120	60	20	625
High-content	75	450	180	120	60	100	545

3.4. Organic compounds without heteroatoms as hydration modulators

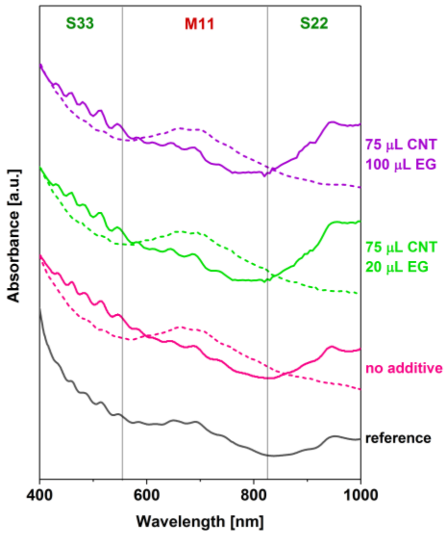

Figure S6 Absorbance spectra of bottom (dashed line) and top (solid line) phases after ATPE carried out in the indicated conditions (with and without ethylene glycol addition).

Table S6. ATPE parameters (ethylene glycol as hydration modulator)

	**Volume [μL]**						
	**CNT 1mg/mL**	**DEX 20%wt**	**PEG 50%wt**	**SC 10%wt**	**SDS 10%wt**	**Ethylene glycol**	**H_2O**
No additive	75	450	180	120	60	–	645
Low-content	75	450	180	120	60	20	625
High-content	75	450	180	120	60	100	545

3.5. Organic compounds with nitrogen atoms as hydration modulators

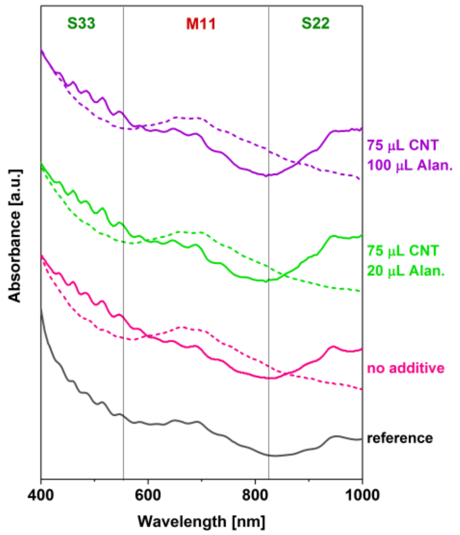

Figure S7 Absorbance spectra of bottom (dashed line) and top (solid line) phases after ATPE carried out in the indicated conditions (with and without alanine addition).

Table S7. ATPE parameters (alanine as hydration modulator)

	**Volume [μL]**						
	**CNT 1mg/mL**	**DEX 20%wt**	**PEG 50%wt**	**SC 10%wt**	**SDS 10%wt**	**Alanine 10%wt**	**H_2O**
No additive	75	450	180	120	60	–	645
Low-content	75	450	180	120	60	20	625
High-content	75	450	180	120	60	100	545

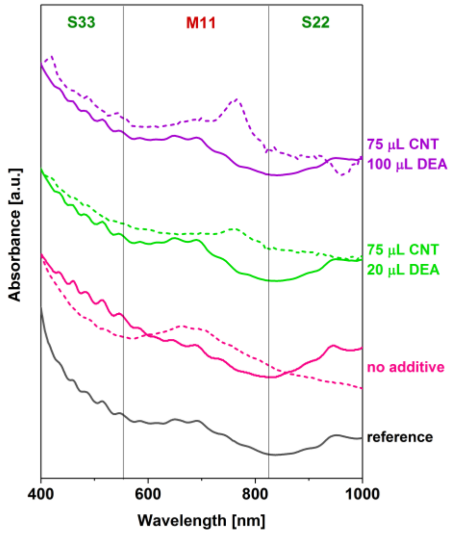

Figure S8 Absorbance spectra of bottom (dashed line) and top (solid line) phases after ATPE carried out in the indicated conditions (with and without diethanolamine addition).

Table S8. ATPE parameters (DEA as hydration modulator)

	**Volume [µL]**						
	**CNT 1mg/mL**	**DEA 20%wt**	**PEG 50%wt**	**SC 10%wt**	**SDS 10%wt**	**DEA**	**H_2O**
No additive	75	450	180	120	60	–	645
Low-content	75	450	180	120	60	20	625
High-content	75	450	180	120	60	100	545

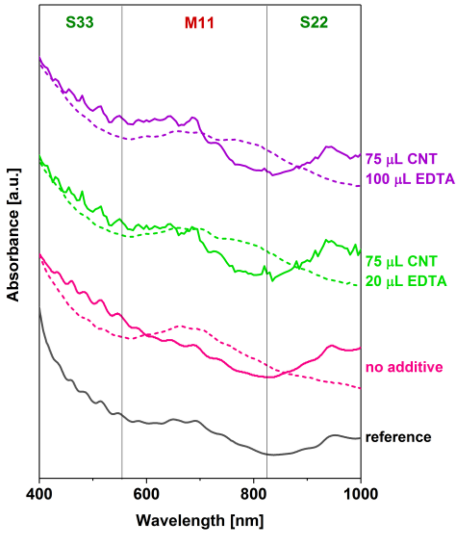

Figure S9 Absorbance spectra of bottom (dashed line) and top (solid line) phases after ATPE carried out in the indicated conditions (with and without EDTA addition).

Table S9. ATPE parameters (EDTA as hydration modulator)

	**Volume [μL]**						
	**CNT 1mg/mL**	**DEX 20%wt**	**PEG 50%wt**	**SC 10%wt**	**SDS 10%wt**	**EDTA 10%wt**	**H_2O**
No additive	75	450	180	120	60	–	645
Low-content	75	450	180	120	60	20	625
High-content	75	450	180	120	60	100	545

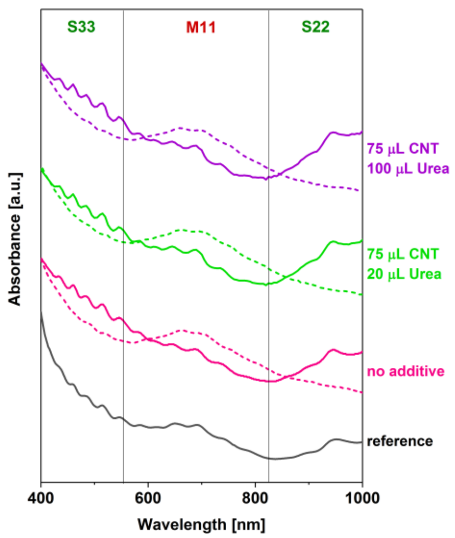

Figure S10 Absorbance spectra of bottom (dashed line) and top (solid line) phases after ATPE carried out in the indicated conditions (with and without urea addition).

Table S10. ATPE parameters (urea as hydration modulator)

	**Volume [μL]**						
	**CNT 1mg/mL**	**DEX 20%wt**	**PEG 50%wt**	**SC 10%wt**	**SDS 10%wt**	**Urea 10%wt**	**H_2O**
No additive	75	450	180	120	60	–	645
Low-content	75	450	180	120	60	20	625
High-content	75	450	180	120	60	100	545

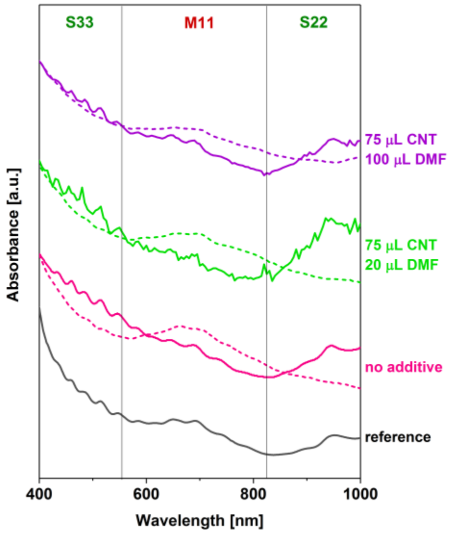

Figure S11 Absorbance spectra of bottom (dashed line) and top (solid line) phases after ATPE carried out in the indicated conditions (with and without DMF addition).

Table S11. ATPE parameters (DMF as hydration modulator)

	Volume [µL]						
	**CNT 1mg/mL**	**DEX 20%wt**	**PEG 50%wt**	**SC 10%wt**	**SDS 10%wt**	**DMF**	**H_2O**
No additive	75	450	180	120	60	–	645
Low-content	75	450	180	120	60	20	625
High-content	75	450	180	120	60	100	545

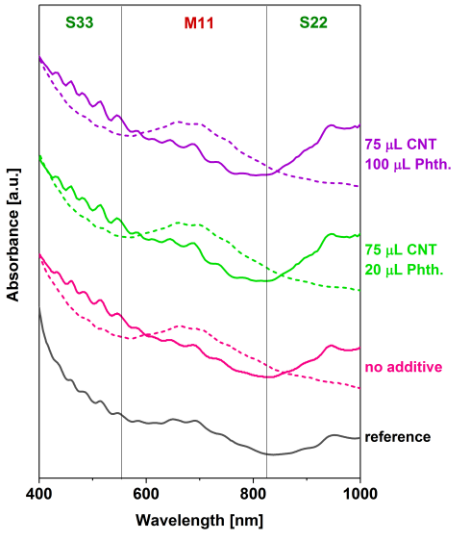

Figure S12 Absorbance spectra of bottom (dashed line) and top (solid line) phases after ATPE carried out in the indicated conditions (with and without phthalimide addition).

Table S12. ATPE parameters (phthalimide as hydration modulator)

	**Volume [μL]**						
	**CNT 1mg/mL**	**DEX 20%wt**	**PEG 50%wt**	**SC 10%wt**	**SDS 10%wt**	**Phth. 10%wt**	**H_2O**
No additive	75	450	180	120	60	–	645
Low-content	75	450	180	120	60	20	625
High-content	75	450	180	120	60	100	545

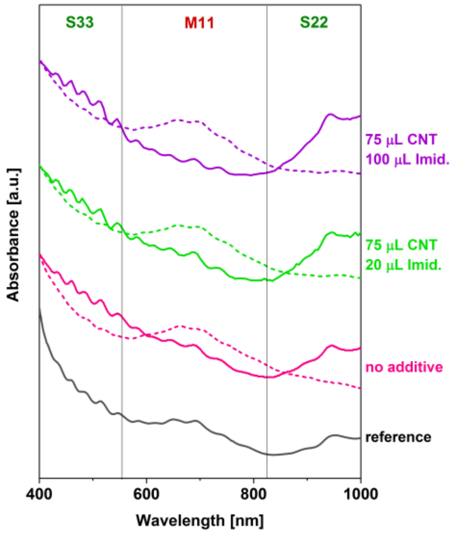

Figure S13 Absorbance spectra of bottom (dashed line) and top (solid line) phases after ATPE carried out in the indicated conditions (with and without imidazole addition).

Table S13. ATPE parameters (imidazole as hydration modulator)

	Volume [µL]						
	**CNT 1mg/mL**	**DEX 20%wt**	**PEG 50%wt**	**SC 10%wt**	**SDS 10%wt**	**Imid. 10%wt**	**H_2O**
No additive	75	450	180	120	60	–	645
Low-content	75	450	180	120	60	20	625
High-content	75	450	180	120	60	100	545

3.6. Organic compounds with sulfur atoms as hydration modulators

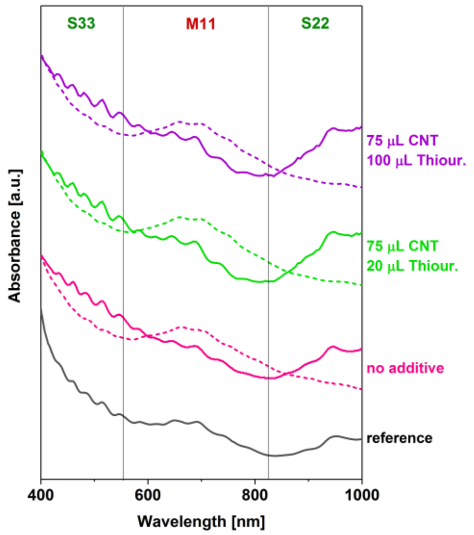

Figure S14 Absorbance spectra of bottom (dashed line) and top (solid line) phases after ATPE carried out in the indicated conditions (with and without thiourea addition).

Table S14. ATPE parameters (thiourea as hydration modulator)

	**Volume [μL]**						
	**CNT 1mg/mL**	**DEX 20%wt**	**PEG 50%wt**	**SC 10%wt**	**SDS 10%wt**	**Thiour. 10%wt**	**H_2O**
No additive	75	450	180	120	60	–	645
Low-content	75	450	180	120	60	20	625
High-content	75	450	180	120	60	100	545

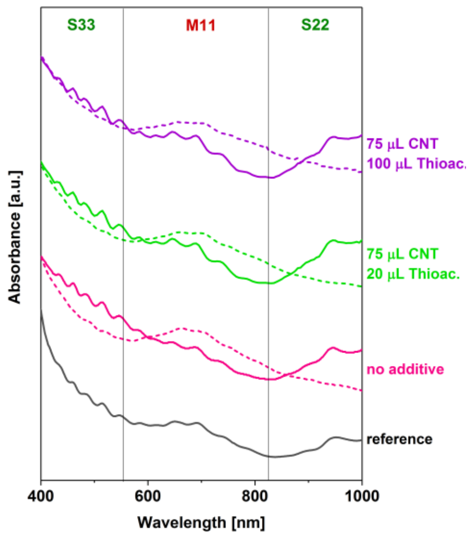

Figure S15 Absorbance spectra of bottom (dashed line) and top (solid line) phases after ATPE carried out in the indicated conditions (with and without thioacetamide addition).

Table S15. ATPE parameters (thioacetamide as hydration modulator)

	**Volume [µL]**						
	**CNT 1mg/mL**	**DEX 20%wt**	**PEG 50%wt**	**SC 10%wt**	**SDS 10%wt**	**Thioac. 10%wt**	**H_2O**
No additive	75	450	180	120	60	–	645
Low-content	75	450	180	120	60	20	625
High-content	75	450	180	120	60	100	545

3.7. Macromolecular species as hydration modulators

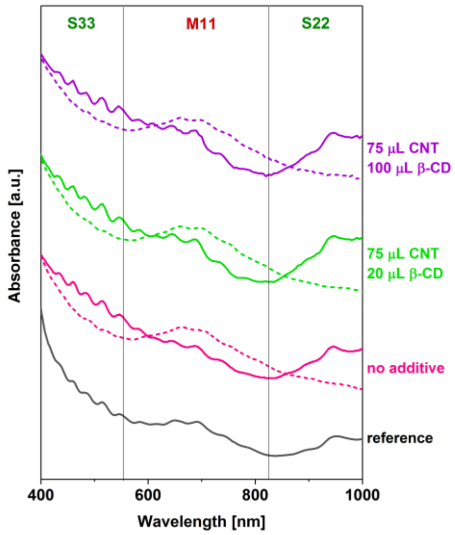

Figure S16 Absorbance spectra of bottom (dashed line) and top (solid line) phases after ATPE carried out in the indicated conditions (with and without β-cyclodextrin addition).

Table S16. ATPE parameters (*β*-CD as hydration modulator)

	**Volume [μL]**						
	**CNT 1mg/mL**	**DEX 20%wt**	**PEG 50%wt**	**SC 10%wt**	**SDS 10%wt**	***β*-CD 10%wt**	**H_2O**
No additive	75	450	180	120	60	–	645
Low-content	75	450	180	120	60	20	625
High-content	75	450	180	120	60	100	545

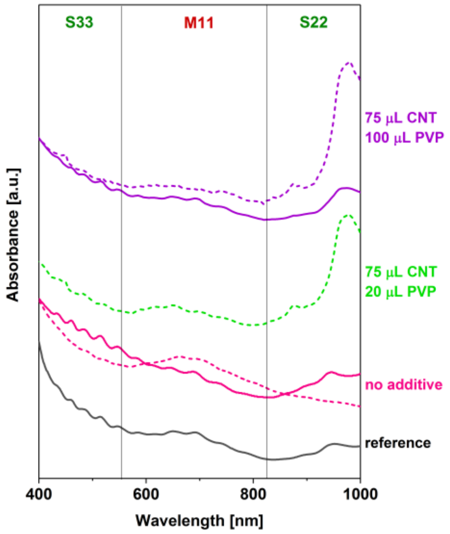

Figure S17 Absorbance spectra of bottom (dashed line) and top (solid line) phases after ATPE carried out in the indicated conditions (with and without PVP addition).

Table S17. ATPE parameters (PVP as hydration modulator)

	**Volume [μL]**						
	**CNT 1mg/mL**	**DEX 20%wt**	**PEG 50%wt**	**SC 10%wt**	**SDS 10%wt**	**PVP 10%wt**	**H_2O**
No additive	75	450	180	120	60	–	645
Low-content	75	450	180	120	60	20	625
High-content	75	450	180	120	60	100	545
